# Multicenter Experience in Robot-Assisted Minimally Invasive Esophagectomy — a Comparison of Hybrid and Totally Robot-Assisted Techniques

**DOI:** 10.1007/s11605-021-05044-8

**Published:** 2021-06-18

**Authors:** Peter P. Grimminger, Julia I. Staubitz, Daniel Perez, Tarik Ghadban, Matthias Reeh, Pasquale Scognamiglio, Jakob R. Izbicki, Matthias Biebl, Hans Fuchs, Christiane J. Bruns, Hauke Lang, Thomas Becker, Jan-Hendrik Egberts

**Affiliations:** 1 Department of General, Visceral and Transplantation Surgery, University Medical Center of the Johannes Gutenberg University Mainz, Langenbeckstrasse 1, D-55131 Mainz, Germany; 2 Department of General, Visceral and Thoracic Surgery, University Medical Center Hamburg-Eppendorf, Hamburg, Germany; 3 Department of Surgery, Charité University Hospital, Berlin, Germany; 4 Department of General, Visceral and Tumor Surgery, University Medical Center Cologne, Cologne, Germany; 5 Department for General, Visceral, Thoracic, Transplantation, and Pediatric Surgery, University Hospital Schleswig Holstein, Kiel, Germany

**Keywords:** Ivor Lewis, Robotic technique, Esophagectomy, Da Vinci Xi, RAMIE

## Abstract

**Background:**

Oncological esophageal surgery has evolved significantly in the last decades. From open esophagectomy over (hybrid) minimally invasive surgery, nowadays, robot-assisted minimally invasive esophagectomy (RAMIE) approaches are applied. Current techniques require an analysis of possible advantages and disadvantages indicating the direction towards a novel gold standard.

**Methods:**

Robot-assisted Ivor Lewis esophagectomies, performed in the period from April 2017 to June 2019 in five German centers (Berlin, Cologne, Hamburg, Kiel, Mainz), were included in this study. Pre-, intra-, and postoperative parameters were assessed. Cases were grouped for hybrid (H-RAMIE) versus totally robot-assisted (T-RAMIE) approaches. Postoperative parameters and complications were compared using risk ratios.

**Results:**

A total of 175 operations were performed as T-RAMIE and 67 as H-RAMIE. Patient age (median age 62 years) and sex (83.1% male) were similarly distributed in both groups. Median duration of esophagectomy was significantly lower in the T-RAMIE group (385 versus 427 min, p < 0.001). The risks of “overall morbidity” (32.0 versus 47.8%; risk ratio [RR], 95% confidence interval (CI): 1.5, 1.1–2.1; p = 0.026), “anastomotic leak” (10.3 versus 22.4%; RR, CI: 2.2, 1.2–4.1; p = 0.020), and “respiratory failure” (1.1 versus 7.5%; RR, CI: 6.5, 1.3–32.9; p = 0.019) were significantly higher in case of H-RAMIE.

**Conclusions:**

In the five participating German centers, T-RAMIE was the preferred procedure (72.3% of operations). In comparison to H-RAMIE, T-RAMIE was associated with a significantly reduced risk of postoperative morbidity, anastomotic leak, and respiratory failure as well as a significantly reduced time necessary for esophagectomy.

## Introduction

Since there is evidence for the superiority of robot-assisted minimally invasive thoraco-laparoscopic esophagectomy over open transthoracic esophagectomy (lower percentage of postoperative complications, shorter hospital stay, and an at least similar oncologic outcome), the robot-assisted approach gained increased interest in recent years.^1^,^2^ Whereas there are only few prospective randomized controlled trials investigating the different techniques for esophageal cancer surgery, most studies are observational, retrospective analyses. However, the undisputed conclusion of the current literature is the advantage of the minimally invasive access.^3^–^6^ The implementation of laparoscopy during Ivor Lewis esophagectomy (= hybrid esophagectomy, HE) was shown to achieve a major benefit concerning pulmonary complications, without compromising overall and disease-free short-term survival in comparison to open surgery.^7^ To date, it has not been proven whether the use of total minimally invasive esophagectomy (MIE) holds advantages over HE regarding the development of postoperative surgical complications and long-term oncological outcome.^8^ Still, MIE was demonstrated advantageous with regard to postoperative pain and rate of pneumonia, with yet similar results for short-term prognosis.^9^ The most recently implemented surgical approach for esophageal cancer is robot-assisted minimally invasive esophagectomy (RAMIE), which — as a major advantage over conventional minimally invasive surgery — facilitates complex minimally invasive procedures by combining a magnified, three-dimensional overview of the intraoperative situs with the possibility of tremor-less tissue dissection with seven degrees of freedom.^10^,^11^ Currently, the position of RAMIE is evaluated in relation to other minimally invasive techniques.^12^ In addition to full RAMIE approaches, also hybrid robot-assisted techniques are applied.^13^ The aim of this multicenter study was to assess the current service-reality for robot-assisted esophagectomy in Germany and to compare postoperative complication rates between totally robot-assisted minimally invasive esophagectomy (T-RAMIE) and hybrid robot-assisted minimally invasive esophagectomy (H-RAMIE) procedures.

## Patients and Methods

### Patients and Centers

Robot-assisted Ivor Lewis esophagectomies, performed in the period from April 2017 to June 2019 in five German centers (Berlin, Cologne, Hamburg, Kiel, Mainz), were included in this retrospective observational study. The study protocol conforms to the ethical guidelines of the 1975 Declaration of Helsinki (6th revision, 2008) as reflected in a priori approval by the institutional human research committees. Informed consent was obtained from all participants.

### Operative Technique: Ivor Lewis Esophagectomy

Two-staged Ivor Lewis esophagectomy (including an abdominal phase with preparation of the gastric conduit and a thoracic phase with esophagectomy and intrathoracic esophagogastric anastomosis) was performed. An insignificant variation of the technique between the participating centers cannot be excluded.

### Abdominal Phase

During the abdominal phase, the lesser omentum was transected upward to the left crus of the diaphragm. The greater gastric curvature was dissected and the stomach mobilized, carefully sparing the right gastroepiploic artery. Abdominal lymphadenectomy usually included lymph nodes at the celiac trunk, along the left gastric and splenic artery and the lesser omentum. The left gastric artery was ligated and transected, allowing for an upward mobilization of the gastric conduit, which was created using a linear stapler.

### Thoracic Phase

For the thoracic phase, after desufflation of the right lung, the parietal pleura was dissected at the anterior side of the esophagus from the diaphragm up to the azygos arch. After ligation and transection of the azygos vein and the thoracic duct, the parietal pleura was dissected along the posterior side of the esophagus. The esophagus was resected en bloc with the surrounding mediastinal and paratracheal lymph nodes. After pulling up the gastric conduit, an esophagogastric anastomosis was created either using a circular stapler, by hand suture or using a combined method.

### Definitions of T-RAMIE and H-RAMIE

Procedures were defined as T-RAMIE, if both the thoracic and abdominal phases were performed robot-assisted. Operations combining a robot-assisted thoracic phase to either a laparoscopic or an open surgical abdominal phase were classified as H-RAMIE.

### Parameters of Assessment

Pre-, intra-, and postoperative parameters were assessed. Pre-operative parameters included basic patient data as sex, age, body mass index (BMI), and comorbidities. Underlying histological entities and neo-adjuvant treatment were registered. Intraoperative parameters included operation time and conversion rate. The registered postoperative data were histological resection margin, number of resected lymph nodes, and stages according to TNM. Operation-associated mortality and postoperative morbidity were assessed. Registered complications were anastomotic leak, conduit necrosis, chyle leak, esophagotracheal fistula, pneumonia, other infections, pneumothorax and respiratory failure, as well as overall morbidity (i.e., number of patients with postoperative complication, with more than one complication possible in one patient). Postoperative pain management, early ambulation, and jenunostomy tube placement were individually handled by the specific centers and were not registered for the present study.

### Statistical Analysis

Data were documented and described using Microsoft Excel (Microsoft Corporation, Redmond, USA). Further analyses were performed using the IBM Statistical Package for Social Science (SPSS) version 23 (IBM Corporation, Armonk, USA). Categorical variables were presented as numbers and percent, and continuous variables as median with range. Cases were grouped for T-RAMIE or H-RAMIE. Comparisons between groups were performed with Fisher’s exact test, Chi-squared test, or Mann–Whitney U test. For postoperative complications, the risk ratio (RR) was calculated, comparing the chance to experience complications for the analyzed groups treated with H-RAMIE (= exposed group) and T-RAMIE. 95% confidence intervals (CI) for RR and p-values resulting from Fisher’s exact test were calculated. Statistical significance was considered with p-values of less than 0.05.

## Results

A total of 175 operations were performed as T-RAMIE (72.3%) and 67 as H-RAMIE (27.7%). Between the groups of H-RAMIE and T-RAMIE, there were no significant differences in patient age (median age: 62 years, range: 22–86), sex (83.1% male), or BMI (median: 26, range: 16–46) (Table 1). Comorbidities were present in 71.1% of cases. Whereas the overall number of patients with comorbidities was similarly distributed between the cohorts, in the H-RAMIE group, there was a significantly higher number of patients with cardiac comorbidity (50.7% versus 23.4%, p < 0.001) and a significantly lower number of patients with vascular comorbidity (3.0% versus 27.4%, p < 0.001) (Table 1). Tumor entities were similarly distributed in both patient groups, with primary adenocarcinoma (82.2%), followed by squamous cell carcinoma (14.5%). Median duration of surgery was significantly shorter in the T-RAMIE group (385 versus 427 min, p < 0.001). Mean duration of surgery for T-RAMIE was 411 min in center I, 326 min in center II, 460 min in center IV, and 463 min in center V (center III did not carry out T-RAMIE procedures). Mean time for H-RAMIE was 326 min in center II, 480 min in center III, 434 min in center IV, and 463 min in center V (center I did not carry out H-RAMIE procedures). In the T-RAMIE group, 4 (2.3%) of esophagogastric anastomoses were hand-sewn, 153 (87.4%) anastomoses stapled, and the remaining 18 (10.3%) were carried out using combined techniques. In the H-RAMIE group, 3 (4.5%) anastomoses were hand-sewn, 52 (77.6%) stapled, and the remaining 12 (17.9%) anastomoses were combined. In center I, 100% of anastomoses were stapled. In center II, 8.9% of anastomoses were hand-sewn, 55.4% stapled, and 35.7% combined. In center III, 35.7% of anastomoses were stapled and 64.3% combined. In center IV, 4.3% of anastomoses were hand-sewn, 93.6% stapled, and 2.1% combined. In center V, 100% of anastomoses were stapled. Conversion to open surgery was necessary in a significantly higher number of cases in the H-RAMIE group (13.4% versus 2.9%, p = 0.004). There were no significant differences in the median number of resected lymph nodes (median: 28, range: 8–81) or in the portion of patients with R0 resection (93.3%, Table 1). Postoperative overall hospital stay was similar for patients treated with T-RAMIE and H-RAMIE (13 vs. 16 days, p = 0.014). Whereas operation-associated mortality was similarly low both for T-RAMIE and H-RAMIE, there were differences in the occurrence of postoperative overall morbidity, which was significantly higher in the H-RAMIE group (47.8% versus 32.0%, p = 0.026). Pneumonia occurred in 37 cases (15.3%), anastomotic leak in 33 cases (13.6%), other infections in 10 cases (4.1%), respiratory failure in 7 cases (2.9%), conduit necrosis and chyle leak equally in 6 cases (2.5%), pneumothorax in 5 cases (2.1%), esophagotracheal fistula in 4 cases (1.7%), and hemorrhage in 2 cases (0.8%, both in T-RAMIE). Calculated per total number of operations, the raw complication rate was 45.5% (110/242).

**Table 1:** Basic data for totally robotic (T-RAMIE) and hybrid robotic (H-RAMIE) approaches

	Total N = 242 (100%)	T-RAMIE N = 175 (72.3%)	H-RAMIE N = 67 (27.7%)	p-value
Age in years (median, range)	62 (22–86)	61 (22–86)	64 (46–79)	0.337 ^c^
Sex				0.340 ^c^
Male (N, %^a^)	201, 83.1	148, 84.6	53, 79.1	
Female (N, %^a^)	41, 16.9	27, 15.4	14, 20.9	
BMI (median, range)	26, 16–46	25, 16–46	25, 16–41	0.310 ^c^
Comorbidity (N, %^a^)	172, 71.1	120, 68.6	52, 77.6	0.205 ^d^
Diabetes (N, %^a^)	27, 11.2	22, 12.6	5, 7.5	0.362 ^d^
Pulmonary comorbidity (N, %^a^)	38, 15.7	25, 14.3	13, 19.4	0.329 ^d^
Cardiac comorbidity (N, %^a^)	75, 31	41, 23.4	34, 50.7	**<0.001** ^**d**^
Vascular comorbidity (N, %^a^)	50, 20.7	48, 27.4	2, 3.0	**<0.001** ^**d**^
Oncologic comorbidity (N, %^a^)	8, 3.3	8, 4.6	0, 0	0.111 ^d^
Neurologic comorbidity (N, %^a^)	12, 5.5	10, 5.7	2, 3.0	0.519 ^d^
No comorbidity (N, %^a^)	70, 28.9	55, 31.4	15, 22.4	0.205 ^d^
Entity				0.245 ^e^
Adenocarcinoma (N, %^a^)	199, 82.2	142, 81.1	57, 85.1	
Squamous cell carcinoma (N, %^a^)	35, 14.5	27, 15.4	8, 11.9	
Other (N, %^a^)	8, 3.3	6,3.5	2, 3.0	
Neoadjuvant therapy				0.336 ^e^
Chemotherapy (N, %^a^)	100, 41.3	76, 43.4	24, 35.8	
Radiochemotherapy (N, %^a^)	101, 41.7	68, 38.8	33, 49.3	
Duration of surgery (median, range)	396, 222–640	385, 222–640	427, 252–579	**<0.001** ^**c**^
Length of hospital stay in days (median, range)	13, 7–92	13, 7–92	16, 10–64	0.014 ^c^
Conversion rate (N, %^a^)	14, 5.8	5, 2.9	9, 13.4	**0.004** ^**d**^
R0 resection (N, %^b^)	223, 93.3	163, 93.7	60, 92.3	0.772 ^d^
N resected lymph nodes (median, range)	28, 8 – 81	28, 8–81	29, 8–54	0.550 ^c^
Histology benign	3	1	2	0.186 ^d^
Histology malignant	239	174	65	0.186 ^d^
T-Stage				0.240 ^e^
T0 (N)	36	27	9	
T1 (N)	43	26	17	
T2 (N)	37	25	12	
T3 (N)	117	92	25	
T4 (N)	6	4	2	
Benign (N)	3	1	2	
N-Stage				0.273 ^e^
N0 (N)	121	83	38	
N1 (N)	44	31	13	
N2 (N)	44	35	9	
N3 (N)	30	25	5	
Benign (N)	3	1	2	
Mortality (N, %^a^)	2, 0.8	2, 1.1	0, 0	1.000 ^d^
Overall morbidity (N, %^a^)	88, 36.4	56, 32.0	32, 47.8	**0.026** ^**d**^

^a^Referred to number of operations per group (total, totally robotic, or hybrid robotic)

^b^Referred to number of malignant tumors per group

^c^Mann-Whitney U test

^d^Fisher’s exact test

^e^Chi-squared test

**Fig. 1:**
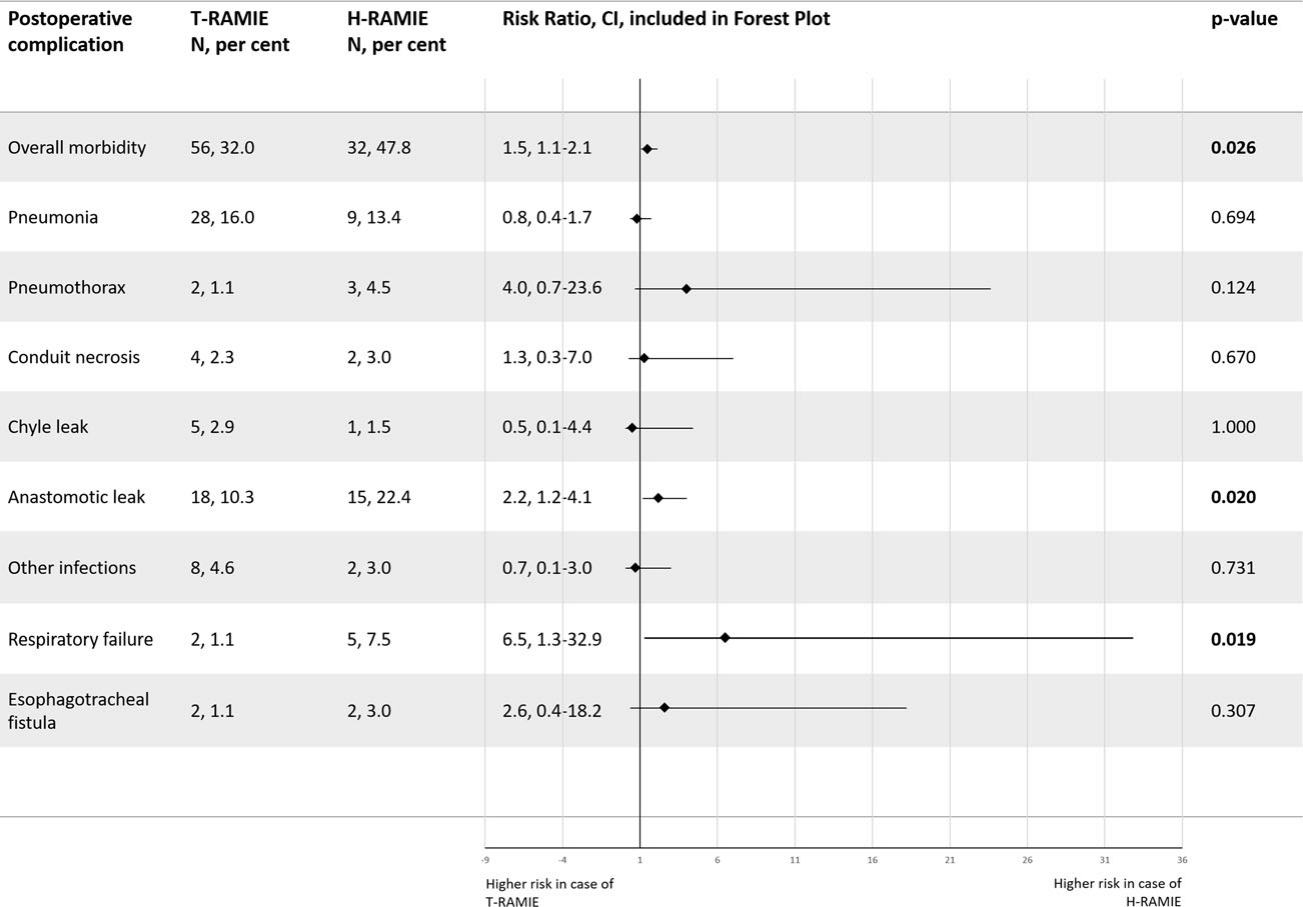
Risk ratios for postoperative complications in relation to the type of approach: totally robotic (T-RAMIE) versus hybrid robotic (H-RAMIE) esophagectomy

The comparison of different postoperative complications showed that the risk of overall morbidity in the H-RAMIE group was 1.5 times as high as in the T-RAMIE group (RR, CI: 1.5 [1.1–2.1], p = 0.026) (Fig. 1). Subjects who underwent H-RAMIE were 2.2 times as likely to develop an anastomotic leak compared to subjects undergoing T-RAMIE (RR, CI: 2.2 [1.2–4.1], p = 0.020). Furthermore, the risk of respiratory failure in the H-RAMIE group was 6.5 times as high as in the T-RAMIE group (RR, CI: 6.5 [1.3–32.9], p = 0.019). Other relevant complications, e.g., esophagotracheal fistula, pneumonia, or development of chyle leak, were similarly rare in both patient groups (Fig. 1).

## Discussion

The present study shows that the majority of patients operated at the participating five German centers were treated with T-RAMIE (72.3% of cases). Still, a relevant number of operations were carried out as H-RAMIE, with the abdominal phase being performed either laparoscopically or by open surgery. The observed overall morbidity after RAMIE procedures, 36.4% (raw complication rate: 45.5%), is below the complication rate of 59%, which was reported by a recent international benchmarking study evaluating service-reality and postoperative morbidity in high-volume centers practicing esophagectomy.^14^ The most common complication in the present cohort was pneumonia (15.3%), followed by anastomotic leak (13.6%). In 2019, Sluis et al. also reported pneumonia and anastomotic leak — as well as cardiac complications — to be the most common adverse events after RAMIE.^2^ The analysis by Sluis et al. illustrated that, except for anastomotic leak, these complications were significantly less frequent in patients treated with RAMIE, compared to open esophagectomy.^11^

In the present analysis, patients belonging to the T-RAMIE and H-RAMIE groups had similar basic conditions, with the absence of significant differences in patient age, sex, and BMI. Also, tumor entities were similarly distributed between both patient groups, with primary adenocarcinoma as the underlying disease and no significant differences concerning the distribution of neoadjuvant treatment. This underlines the relevance of the choice of the surgical technique for the occurrence of significantly different postoperative complication rates: patients receiving H-RAMIE had a significantly higher risk of postoperative morbidity, especially for anastomotic leak (RR 2.2) and respiratory failure (RR 6.6). These results illustrate the superiority of the T-RAMIE approach. Reasons for the advantage of T-RAMIE in terms of the development of anastomotic leaks may lie in several issues such as a learning curve effect and also surgical procedure-related factors like the reduction of shearing forces during the preparation of the gastric conduit as well as during the thoracic phase, which is facilitated by the dissection with seven degrees of freedom.^15^,^16^ The worse outcome in the H-RAMIE group concerning anastomotic leak (10.3% for T-RAMIE vs. 22.4% for H-RAMIE) is also accountable for the relatively high overall leak rate of 13.6% in the present study, which lays above the rate of 9.4%, reported by Zhai et al. for minimally invasive Ivor Lewis esophagectomy^17^ or the rate of 12% reported by Tagkalos et al. for robot-assisted minimally invasive Ivor Lewis esophagectomy.^18^ Yet, the present overall leak rate is below the rate of 19%, reported by Sluis et al. in robot-assisted minimally invasive thoraco-laparoscopic esophagectomy.^16^ Furthermore, the robot-assisted performance of the thoracic phase allows for a higher accuracy in performing lymph node dissection (especially in the upper mediastinum) when compared to, e.g., the MIE approach.^19^–^21^ The median lymph node count in the present study was 28, without significant differences between the T-RAMIE and H-RAMIE groups, which is comparable to the mean lymph node yield reported by Sluis et al. in 2019.^2^ Another advantage of T-RAMIE was the significantly reduced operation time in comparison to H-RAMIE. As a reason for this observation, the use of the robotic system for the abdominal phase in cases of T-RAMIE might contribute to a better practice for surgeons in robotic training, potentially culminating in a steeper learning curve for the performance of the thoracic part and, therefore, a shorter overall operation time. However, in addition to the influence of the method itself, also the mean operation times at the participating centers may have influenced this result.

The significantly elevated risk of postoperatively registered respiratory failure in the H-RAMIE group might have been biased partly by an unequal distribution of cardiac comorbidities in the cohorts. Still, the risk of respiratory failure, which in the H-RAMIE group was 6.5 times as high as in the T-RAMIE group, is considerable. Especially in cases of H-RAMIE which included an open abdominal phase instead of a minimally invasive approach, it is in line with the literature that respiratory complications are more likely to be observed.^7^ Another important advantage of T-RAMIE was the significantly lower rate of conversion to open surgery. A potential reason might be the training effect of the surgeons performing T-RAMIE or more robot-assisted operations as such, as the more profound acquaintance with the technique potentially facilitates a robotic complication management, which otherwise would require conversion to open surgery. The higher conversion rate in the H-RAMIE group might additionally have acted as an important influencing factor: since H-RAMIE (conversion rate 13.4%) was associated with greater operation trauma, an elevation in the postoperative morbidity rate is plausible. Of note is the similarity in the oncological outcome between the T-RAMIE and H-RAMIE cohorts of the present study, as measured by the number of resected lymph nodes (median 28 lymph nodes) and the number of R0 resections (93.3%). Postoperative mortality was 0.8%, without significant differences between the cohorts. Na et al., who compared T-RAMIE and H-RAMIE (= robot-assisted esophagectomy with abdominal procedures performed by laparotomy) procedures in a propensity-matched analysis, found similarly favorable 2-year survival rates for T-RAMIE (86.2%) and H-RAMIE (77.6%).^13^ With a higher overall complication rate of 63.3% (versus 36.4% in the present study); however, Na et al. did not observe significant differences between complication rates neither in the matched nor in the unmatched comparison between T-RAMIE and H-RAMIE.^13^

In addition to the advantages of T-RAMIE over H-RAMIE, which were illustrated by this study, there is an economic aspect to be taken into consideration: the use of the robot-attached energy devices during both the abdominal and thoracic phases allows for the saving of the extra cost of single-use handheld energy devices.

## Conclusion

In the period from April 2017 to June 2019, the majority of robot-assisted esophagectomies in the cohort of the German Centers Berlin, Cologne, Hamburg, Kiel, and Mainz were performed as T-RAMIE. In comparison to H-RAMIE, the use of T-RAMIE was associated with a significantly reduced risk of postoperative morbidity, anastomotic leak, and respiratory failure as well as a significantly reduced time necessary for esophagectomy. Short-time oncological outcome (as measured by number of R0 resections and median number of resected lymph nodes) was similar for T-RAMIE and H-RAMIE.
